# Prognostic Value of Abnormal Liver Function Tests After Mechanical Thrombectomy for Acute Ischemic Stroke

**DOI:** 10.3389/fneur.2021.670387

**Published:** 2021-07-28

**Authors:** Kangmo Huang, Mingming Zha, Lulu Xiao, Jie Gao, Juan Du, Min Wu, Qingwen Yang, Rui Liu, Xinfeng Liu

**Affiliations:** ^1^Department of Neurology, Affiliated Jinling Hospital, Medical School of Nanjing University, Nanjing, China; ^2^Department of Neurology, Jinling Hospital, Medical School of Southeast University, Nanjing, China; ^3^Department of Neurology, Jinling Hospital, The First School of Clinical Medicine, Southern Medical University, Nanjing, China

**Keywords:** liver function tests, ischemic stroke, thrombectomy, prognosis, observational study

## Abstract

**Objective:** To determine the clinical significance of post-procedural abnormal liver function test (ALFT) on the functional outcomes at 90 days in acute ischemic stroke (AIS) treated with mechanical thrombectomy (MT).

**Methods:** In this retrospective observational study, patients with AIS undergoing MT were enrolled from the Nanjing Stroke Registry Program and the multicenter Captor trial. A favorable outcome was defined as a modified Rankin Scale score 0–2 at 90 days. Predictive models were established by multivariable logistic regression. Improved predictive value of models was assessed by continuous net reclassification improvement (NRI) and integrated discrimination improvement (IDI). In addition, multivariable logistic regression and restricted cubic spline were used to analyze dose–response correlations between the severity of ALFT and prognosis.

**Results:** Among 420 patients enrolled, 234 (55.7%) patients were diagnosed as post-procedural ALFT after MT. Patients with post-procedural ALFT had higher National Institute of Health Stroke Scale score on admission (median, 18 vs. 15, *p* < 0.001) and more pneumonia (65.4 vs. 38.2%, *p* < 0.001) than those without post-procedural ALFT. Post-procedural ALFT, rather than preprocedural ALFT, was independently associated with favorable outcome (adjusted odds ratio, 0.48; 95% CI 0.28–0.81; *p* = 0.006). The improvement of predictive model after adding post-procedural ALFT was significant [continuous NRI (value, 0.401; *p* < 0.001), IDI (value, 0.013; *p* < 0.001)]. However, the restricted cubic spline indicated no evidence of a dose–response relationship between the severity of post-procedural ALFT and prognosis.

**Conclusions:** In AIS patients treated by MT, post-procedural ALFT was associated with more severe stroke and served as an independent predictor of worse prognosis at 90 days.

## Introduction

Mechanical thrombectomy (MT) is a well-established treatment for acute ischemic stroke (AIS) caused by large vessel occlusions (LVO) (1). However, regardless of the proven validity of MT, only approximately 40% of patients undergoing MT could achieve functional independence at 90 days (1, 2). Despite the growing attention, factors affecting clinical outcomes have not been fully recognized yet. Conventional factors including age, baseline disability, history of hypertension, and hyperglycemia were known to be associated with worse prognosis in AIS patients treated with MT (3). Besides, radiological markers of chronic brain damage, such as cortical microinfarcts (4), leukoaraiosis (5), brain atrophy (6), and cerebral microbleeds (7), have recently been identified as risk factors of poor clinical outcomes. Comorbidities and complications such as malignant brain edema (8), pneumonia (9), and renal dysfunction (10) were reported as potential risk factors for worse prognosis, too.

Liver is a vital organ with essential biosynthetic and metabolic functions. It was reported that abnormal liver function test (ALFT) was associated with higher mortality risk in critically ill patients (11–14). ALFTs are extremely common in AIS patients, with an incidence of about 40% (15–18). A prospective study showed more severe stroke and worse outcomes in AIS patients with non-alcoholic fatty liver disease (15). However, there are scarce data regarding the association between ALFT and clinical outcomes in AIS-LVO patients undergoing MT.

The aims of this study were to (1) determine the association between periprocedural ALFT and the clinical outcomes, and (2) assess the potential dose–response relationship between liver function test levels and prognosis of AIS-LVO patients undergoing MT.

## Methods

### Data Resources

Patients were screened from the Nanjing Stroke Registry Program (NSRP, between January 2014 and December 2019) and the Captor trial (a multi-center clinical trial between March 2018 and July 2019, register code: ChiCTR1900025256). Detailed descriptions of NSRP had been published elsewhere (19). The Captor trial was aimed to compare the Captor stent retriever with the Solitaire FR device for rapid flow restoration in AIS-LVO. The non-inferiority of the Captor retrievable stent was proved after enrolling 245 patients from 16 comprehensive stroke centers in China.

### Inclusion and Exclusion Criteria

The key inclusion criteria were as follows: (1) age ≥18 years; (2) National Institute of Health Stroke Scale (NIHSS) score on admission ≥6; (3) occlusion of the intracranial large vessel (defined as diameter ≥2 mm) proved by CT angiography, magnetic resonance angiography, or digital subtraction angiography; (4) completion of groin-puncture within 8 h since stroke onset.

The key exclusion criteria were as follows: (1) active hemorrhage or hemorrhagic tendency; (2) severe organic diseases such as heart, lung, liver, and kidney failure; (3) occlusion attributed to arterial dissection or arteritis. The Ethics Committee of Jinling Hospital and each participating center approved this study according to the Declaration of Helsinki. The requirement for written informed consent was waived because of its retrospective nature.

### Data Collections

Baseline demographic parameters, medical histories, laboratory tests, the NIHSS scores, and radiographic evaluation were obtained. The treatment profiles were also gathered, including intravenous thrombolysis (IVT), number of retrieval attempts, final reperfusion status, and procedural time. IVT was administrated within 4.5 h of stroke onset after excluding contraindications. Retrieval attempts were recommended no more than three times, but it was at the discretion of the interventionalists. Reperfusion was evaluated with the modified Thrombolysis In Cerebral Ischemia scale (mTICI) (20). Successful reperfusion was defined as the mTICI scale score of 2b or 3.

Post-procedural NIHSS scores of all patients were assessed by attending neurologists. Follow-up imaging examinations were performed within 24 ± 6 h after the procedure. Symptomatic intracranial hemorrhage (SICH) was defined as imaging evidence of intracranial hemorrhage (ICH) with an increase of ≥4 points on the NIHSS within 24 h. On day 7 ± 2 or hospital discharge if earlier, assessments of neurological severity were performed on the subjects again.

Post-procedural laboratory tests were carried out routinely in the acute phase (7 ± 2 days after MT) and/or in case of clinical deterioration. Liver function was assessed according to tests of serum aspartate aminotransferase [upper limit of normal (ULN): 40 U/L], alanine aminotransferase (ULN: 40 U/L), total bilirubin (ULN: 21 mmol/L), and direct bilirubin (ULN: 10 mmol/L). ALFT was defined as any elevated value above the ULN (21). Multiple_max_ was defined as the maximum multiple of ULN in the four variables (aspartate aminotransferase, alanine aminotransferase, total bilirubin, and direct bilirubin) during hospitalization after MT.

Follow-up was performed by outpatient clinical visits or telephone contacts to record the modified Rankin Scale (mRS) score at 90 ± 14 days. A favorable outcome was defined as mRS 0–2 at 90 days, and an excellent outcome was referred to as mRS 0–1 at 90 days.

### Statistical Analysis

Median imputation was performed to account for missing values [white blood cell: 6 (1.4%); creatinine: 9 (2.1%); glucose: 11 (2.6%); activated partial thromboplastin time: 17 (4.0%); partial thromboplastin time: 13 (3.1%); international normalized ratio: 13 (3.1%)].

Frequency (percentage) was used for categorical variables. Mean (SD) was adopted for continuous variables with normal distribution and median (interquartile range, IQR) for non-normal variables.

Univariate analysis was conducted with Student *t*-test, Mann–Whitney *U*, χ^2^ test, or Fisher exact test as appropriate. Confounding factors and other variables with a statistical trend (*p* ≤ 0.1) in the univariate analysis were included in multivariate logistic regression analysis (forward selection) to develop a basic model for the favorable prognosis. Composite model was established by factors of basic model and IVT, ASITN/SIR, recanalization, and SICH. Besides, the predictive value of adding preprocedural or post-procedural ALFT into the basic model was estimated by the area under curve (AUC). The improvements on the basic model were assessed by the continuous net reclassification index (NRI) and integrated discrimination improvement (IDI) of which values above 0 were regarded as significant (22). Multiple_max_ was categorized based on quartiles to evaluate the association between Multiple_max_ and prognosis. The pattern of the potential dose–response relationship between Multiple_max_ and prognosis was revealed using a restricted cubic spline with 3 knots after adjusting for confounders. Odds ratio (OR) was reported with a 95% CI.

Subgroup analyses were performed to detect the heterogeneity in the effect of post-procedural ALFT on prognosis. Sensitivity analyses were conducted to explore the effect of ALFTs at different periods on prognosis. A two-tailed *p* ≤ 0.05 was considered statistically significant. Statistical analyses were performed with the SPSS software package, version 26 (IBM, Armonk, NY) and R statistical software 3.6.3 [R Core Team (2020). R: A language and environment for statistical computing. R Foundation for Statistical Computing, Vienna, Austria. URL https://www.R-project.org/].

## Results

### Clinical Characteristics

The flowchart of this study is illustrated in Supplementary Figure 1. The demographic characteristics of all participants are displayed in Table 1. Of the 420 patients enrolled in this study, men accounted for 60.7% and the median age was 68 years. In total, 234 (55.7%) subjects presented as post-procedural ALFT, and 186 (44.3%) had normal liver function after MT.

**Table 1:** Clinical characteristics in the ALFT group and the non-ALFT group.

	**All patients** **(***n*** = 420)**	**ALFT** **(***n*** = 234)**	**Non-ALFT** **(***n*** = 186)**	***P*** **-value**
**Demographics**				
Age, median (IQR)	68 (58–76)	69 (58–76)	68 (58–75)	0.745
Male, *n* (%)	255 (60.7)	150 (64.1)	105 (56.5)	0.111
SBP, mean (SD)	140 (22)	140 (23)	140 (20)	0.660
**Medical history**, ***n*****(%)**				
Hypertension	258 (61.4)	152 (65.0)	106 (57.0)	0.096
Diabetes	77 (18.3)	45 (19.2)	32 (17.2)	0.594
Hyperlipidemia	20 (4.8)	14 (6.0)	6 (3.2)	0.188
Coronary heart disease	78 (18.6)	49 (20.9)	29 (15.6)	0.161
Stroke	71 (16.9)	37 (15.8)	34 (18.3)	0.503
Smoke	128 (30.5)	74 (31.6)	54 (29.0)	0.567
Atrial fibrillation	184 (43.6)	112 (47.9)	71 (38.2)	0.047
Preprocedural ALFT ^*^	124 (29.5)	94 (40.2)	30 (16.1)	<0.001
Baseline NIHSS score, median (IQR)	16 (13–21)	18 (13–23)	15 (12–19)	<0.001
**Occlusion site**, ***n*****(%)**				
Intracarotid artery	137 (32.6)	84 (35.9)	53 (28.5)	0.108
Middle cerebral artery	236 (56.2)	115 (49.1)	121 (65.1)	0.001
Anterior cerebral artery	10 (2.4)	6 (2.6)	4 (2.2)	0.782
Vertebrobasilar artery	51 (12.1)	35 (15.0)	16 (8.6)	0.048
**TOAST**, ***n*****(%)**				0.003
Large artery atherosclerosis	130 (31.0)	77 (32.9)	53 (28.5)	
Cardio-embolism	186 (44.3)	114 (48.7)	72 (38.7)	
Others	104 (24.8)	43 (18.4)	61 (32.8)	
**Laboratory findings, median (IQR)**				
WBC, 10^9^/L	8.39 (6.69–10.4)	8.67 (6.91–10.60)	7.95 (6.45–9.90)	0.019
Glu, mmol/L	7.38 (6.20–8.49)	7.50 (6.40–8.85)	7.04 (6.00–8.20)	0.035
eGFR, ml/min/1.73 m^2^	92 (76–103)	91 (74–103)	92 (79–103)	0.911
APTT, s	27.0 (23.7–32.9)	27.0 (23.2–32.3)	27.0 (24.4–33.7)	0.104
PT, s	12.2 (11.3–13.4)	12.3 (11.2–13.2)	12.2 (11.2–13.5)	0.699
INR	1.03 (0.97–1.10)	1.03 (0.97–1.11)	1.03 (0.97–1.18)	0.168
**Operation procedures**				
IVT, n (%)	133 (31.7)	80 (34.2)	53 (28.5)	0.213
ASITN/SIR, median (IQR)	1 (1–2)	1 (1–2)	1 (1–2)	0.784
mTICI 2b−3, *n* (%)	345 (82.1)	186 (79.5)	159 (85.5)	0.111
Operation time, min, median (IQR)	97 (69–136)	97 (71–144)	95 (67–130)	0.329
SICH, *n* (%)	35 (8.3)	28 (12.0)	7 (3.8)	0.003
Pneumonia, *n* (%)	224 (53.3)	153 (65.4)	71 (38.2)	<0.001
NIHSS score at 24 h, median (IQR)	15 (8–22)	19 (11–25)	11 (6–16)	<0.001
**Clinical outcomes**				
Mortality at 7 days, *n* (%)	43 (10.2)	28 (12.0)	15 (8.1)	0.190
7-day NIHSS score, median (IQR)	11 (4–20)	14 (8–24)	6 (2–14)	<0.001
Excellent prognosis, *n* (%)	113 (26.9)	36 (15.4)	77 (41.4)	<0.001
Favorable prognosis, *n* (%)	168 (40.0)	62 (26.5)	106 (57.0)	<0.001
Mortality at 90 days, *n* (%)	97 (29.1)	68 (29.1)	29 (15.6)	0.001
90-day mRS score, median (IQR)	3 (1–5)	4 (2–6)	2 (1–4)	<0.001

* *For 26 patients without any results of preprocedural liver function tests, they were considered to be free of preprocedural ALFT*.

*ALFT, abnormal liver function test; IQR, interquartile range; SBP, systolic blood pressure; NIHSS, National Institute Health Stroke Scale; TOAST, trial of org 10,172 in acute stroke treatment; WBC, white blood cell; eGFR, estimated glomerular filtration rate; APTT, activated partial thromboplastin time; PT, partial thromboplastin time; INR, international normalized ratio; IVT, intravenous thrombolysis; ASITN/SIR, American Society of Interventional and Therapeutic Neuroradiology/Society of Interventional Radiology System; mTICI, modified Thrombolysis In Cerebral Ischemia; SICH, symptomatic intracranial hemorrhage; mRS, modified Rankin scale*.

Patients were divided into two groups (the ALFT group and the non-ALFT group) according to the post-procedural ALFT. Medical histories were balanced between the two groups. The ALFT group had a significantly larger proportion of preprocedural ALFT (40.2 vs. 16.1%, *p* < 0.001). Higher NIHSS score on admission (median, 18 vs. 15, *p* < 0.001), higher white blood cell count (*p* = 0.019), higher glucose level (*p* = 0.035), more pneumonia (65.4 vs. 38.2%, *p* < 0.001), and more SICH (12.0 vs. 3.8%, *p* < 0.001) were detected in the ALFT group than the non-ALFT group. The ratio of IVT pretreatment (34.2 vs. 28.5%, *p* = 0.213) was similar between patients with normal and abnormal liver function. The comparisons between the two groups on median NIHSS scores at 24 h (19 in the ALFT group vs. 11 in the non-ALFT group, *p* < 0.001) and 7 days (14 in the ALFT group vs. 6 in the non-ALFT group, *p* < 0.001) showed similar trends with that of the admission NIHSS score (Table 1).

### Clinical Outcomes

The mortality rate at 7 days was comparable between the two groups (*p* = 0.190). More deaths occurred in the ALFT group at 90 days (29.1 vs. 15.6%, *p* = 0.001, Table 1).

**Figure 1:**
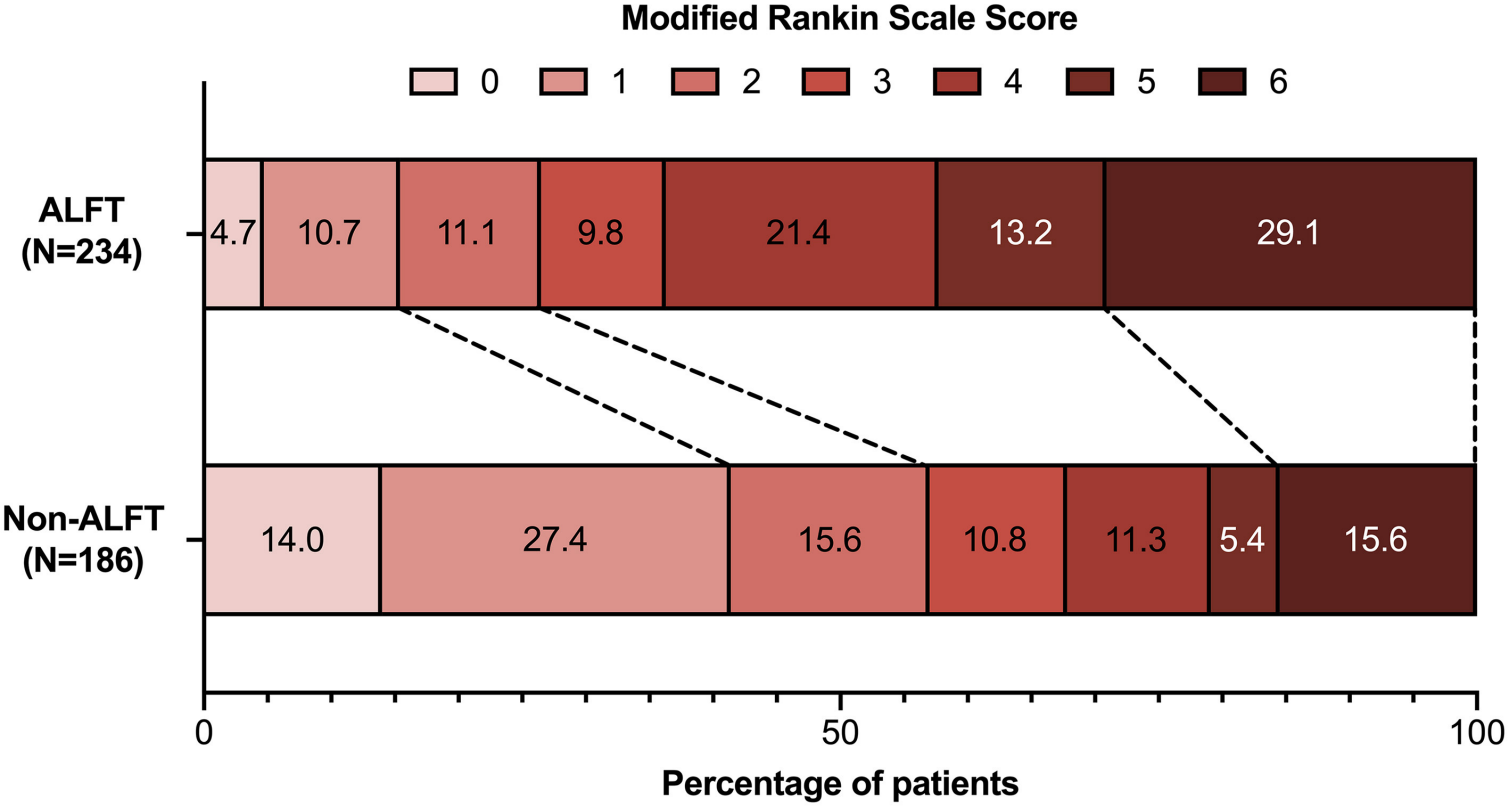
Comparisons of modified Rankin scale scores at 90 days between the ALFT group and the non-ALFT group. ALFT, abnormal liver function test.

In total, 168 (40.0%) patients ranked 0–2 on mRS score at 90 days after MT, and the proportion of excellent prognosis was 26.9%. Compared with the non-ALFT subjects, the ALFT group had a lower percentage of favorable prognosis (26.5 vs. 57.0%, *p* < 0.001) and excellent prognosis (15.4 vs. 41.4%, *p* < 0.001) at 90 days (Figure 1). The density plot of *log-transformed* Multiple_max_ distribution for dichotomous prognosis is shown in Supplementary Figure 2. Smaller Multiple_max_ was detected in patients achieving better prognosis.

### Multivariable Analyses

The basic model for predicting favorable prognosis was developed after adjusting for confounding factors and variables with a statistical trend (*p* ≤ 0.1) in the univariate analysis. Finally, age [OR (95% CI): 0.96 (0.94–0.98), *p* < 0.001], the NIHSS score at 24 h [OR (95% CI): 0.85 (0.82–0.88), *p* < 0.001], pneumonia [OR (95% CI): 0.43 (0.25–0.72), *p* = 0.002], and glucose level [OR (95% CI): 0.81 (0.71–0.92), *p* = 0.001] on admission were included in the basic model (Table 2).

**Table 2:** Multivariable logistic regression for predicting favorable outcomes.

**Variable**	**OR (95% CI)**	***P*-value**
**Basic model** ^*^		
Age	0.96 (0.94–0.98)	<0.001
24-h NIHSS score	0.85 (0.82–0.88)	<0.001
Pneumonia	0.43 (0.25–0.72)	0.002
Glucose	0.81 (0.71–0.92)	0.001
**Basic model** **+** **preprocedural ALFT**		
Preprocedural ALFT	1.615 (0.902–2.891)	0.107
**Basic model** **+** **post-procedural ALFT**		
Post-procedural ALFT	0.48 (0.28–0.81)	0.006
**Composite model** ^#^ **+** **post-procedural ALFT**		
Post-procedural ALFT	0.45 (0.26–0.78)	0.004

* *Basic model was established by confounders and variables with a statistical trend (p ≤ 0.1) in the univariate analysis using the stepwise forward method*.

# *Composite model was established by factors of basic model and IVT, ASITN/SIR, recanalization, and SICH*.

*OR, odds ratio; NIHSS, National Institute of Health Stroke Scale; IVT, intravenous thrombolysis; ALFT, abnormal liver function test; IVT, intravenous thrombolysis; ASITN/SIR, American Society of Interventional and Therapeutic Neuroradiology/Society of Interventional Radiology System; SICH, symptomatic intracranial hemorrhage*.

An improved model was established after adding ALFT into the basic model. As shown in the improved model, post-procedural ALFT had a significant correlation [OR (95% CI): 0.48 (0.28–0.81), *p* = 0.006] with mRS 0–2 at 90 days after adjusting for the factors involved in the basic model (Table 2). Compared with the basic model, the predictive value of the improved model on favorable prognosis showed a significant improvement when assessed by AUC (basic model: 0.875; improved model: 0.881, *p* = 0.007), continuous NRI (value: 0.401, *p* < 0.001), and IDI (value: 0.013, *p* < 0.001), as shown in Table 3. In addition, preprocedural ALFT was not a statistically significant predictor of clinical prognosis (all *p*-values > 0.05).

**Table 3:** Comparison of basic models and models adding ALFT for predicting favorable prognosis and excellent prognosis.

	**AUC**	***P*-value**	**Continuous NRI (95% CI)**	***P*-value**	**IDI (95% CI)**	***P*-value**
Basic model	0.875	–	Reference	–	Reference	–
Preprocedural ALFT	0.878	0.106	0.068 (−0.112, −0.247)	0.460	0.005 (−0.016, −0.012)	0.133
Post-procedural ALFT	0.881	0.007	0.401 (0.211, 0.591)	<0.001	0.013 (0.013, 0.045)	<0.001

*ALFT, abnormal liver function test; AUC, area under curve; NRI, net reclassification improvement; IDI, integrated discrimination improvement*.

**Figure 2:**
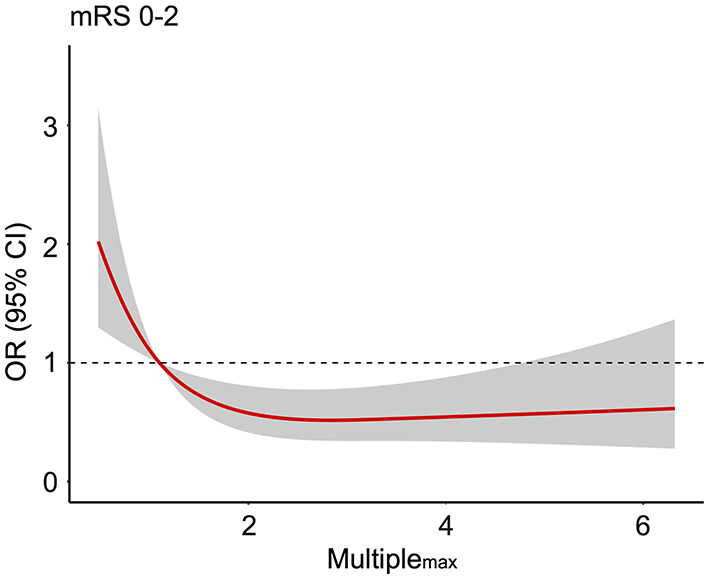
The association between Multiple_max_ and the odds ratio for favorable prognosis (90-day modified Rankin scale scores 0–2). Adjusted for age, median NIHSS score at 24 h, pneumonia, and glucose level. mRS, modified Rankin scale; OR, odds ratio; NIHSS, National Institute of Health Stroke Scale.

The role of Multiple_max_ in predicting favorable prognosis was also assessed in multivariable analysis. After adjusting for age, pneumonia, glucose level, and median NIHSS score at 24 h, Multiple_max_ remained an independent predictive factor of favorable prognosis (*p* = 0.019, Table 4). To explore potential dose–response association, the restricted cubic spline was drawn with three knots after adjusting for covariates mentioned previously. Interestingly, increasing Multiple_max_ was no longer lower odds of favorable prognosis (Figure 2) further when Multiple_max_ was more than 2.

**Table 4:** Multiple_max_ for predicting favorable prognosis (modified Rankin Scale 0–2).

	**mRS 0–2** ***n*** **(%)**	**Univariable analysis** **OR (95% CI)**	***P*** **-value**	**Multivariable analysis** ^*^ **OR (95% CI)**	***P*** **-value**
Multiple_max_, quartiles			<0.001		0.019
(0–0.73) UNL	68 (63.6)	Reference		Reference	
(0.73–1.10) UNL	48 (44.9)	0.47 (0.27–0.81)	0.006	0.55 (0.28–1.07)	0.080
(1.10–1.80) UNL	32 (31.7)	0.27 (0.15–0.47)	<0.001	0.37 (0.18–0.76)	0.007
> 1.80 UNL	20 (19.0)	0.14 (0.07–0.25)	<0.001	0.35 (0.16–0.75)	0.007

* *Adjusted for age, dichotomous NIHSS score at 24 h (divided by median), pneumonia, and glucose level*.

*mRS, modified Rankin Scale; OR, odds ratio; UNL, upper limit of normal*.

### Subgroup Analyses and Sensitivity Analyses

**Figure 3:**
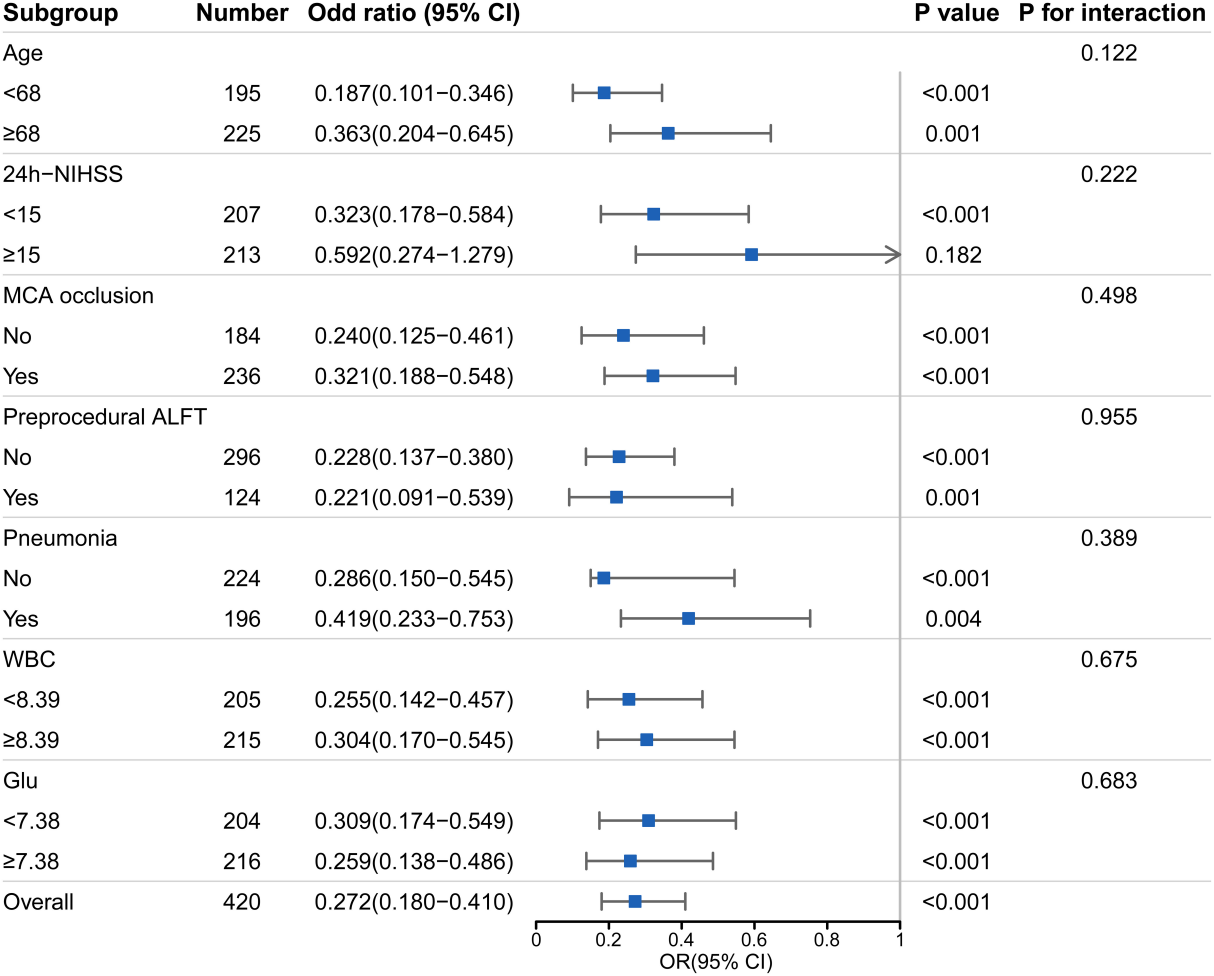
Subgroup analyses of the association between post-procedural ALFT and the favorable prognosis. This forest plot shows the odds ratio for favorable prognosis (90-day modified Rankin scale scores 0–2) of post-procedural ALFT in subgroups. NIHSS, National Institute Health Stroke Scale; MCA, middle cerebral artery; ALFT, abnormal liver function; WBC, white blood cell; mRS, modified Rankin scale; OR, odds ratio.

A dominant tendency toward a lower proportion of favorable prognosis in the ALFT group was elucidated in the subgroup analysis (all *p*-values < 0.01, except for subjects who had NIHSS score ≥15) (Figure 3). Of note, no significant interaction was detected between post-procedural ALFT and these clinical features on the favorable prognosis.

**Figure 4:**
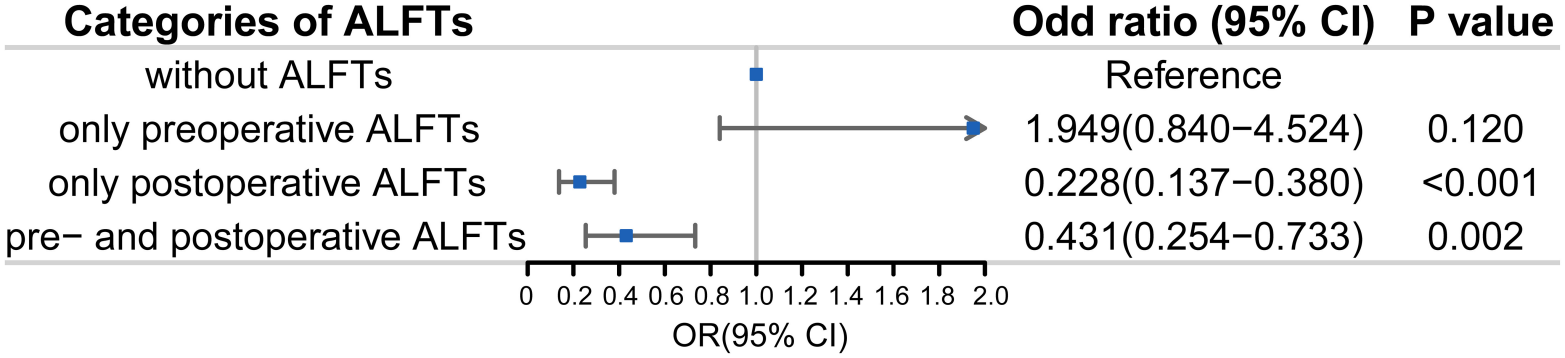
Association between ALFTs at different periods and the favorable prognosis in patients with AIS. ALFT, abnormal liver function; OR, odds ratio.

In sensitivity analyses, patients with post-procedural ALFTs alone had a lower proportion of favorable prognosis [OR (95% CI): 0.23 (0.14–0.38), *p* < 0.001] than patients with preprocedural ALFTs alone [OR (95% CI): 1.94 (0.84–4.52), *p* = 0.120] (Figure 4). They even had less favorable prognoses than patients with both pre- and post-procedural ALFTs [OR (95% CI): 0.23 (0.14–0.38) vs. 0.43 (0.25–0.73)].

## Discussion

In this multi-center retrospective study, 55.7% of all AIS patients undergoing MT were identified as post-procedural ALFT. Patients with post-procedural ALFT had more severe stroke, and higher WBC count and blood glucose values on admission. The presence of post-procedural ALFT, rather than the preprocedural ALFT, was independently associated with a decreased proportion of favorable outcomes at 90 days. However, there was no evidence of a dose–response relationship between the severity of post-procedural ALFT and functional prognosis.

The prevalence of post-procedural ALFT in patients with AIS in our study was 55.7%, higher than the previously reported frequency (about 40%) in AIS patients (15–18). Post-procedural ALFT may be a consequence of neuroendocrine dysregulation after stroke onset (23). Focal brain ischemia could induce systemic pathophysiologic reactions and contribute to hepatic inflammatory and apoptotic activation (24). Stroke-induced catecholamine surge promotes the endoplasmic reticulum stress and impairs hepatic insulin signaling (25). Moreover, elevated bilirubin and liver enzyme levels may also be attributed to the treatment measures of AIS, such as antibiotic drugs, statin, and endovascular therapy (21, 26). Therefore, metabolic imbalances and the reflection of treatment during hospitalization are conjectured to be major mechanisms of post-procedural ALFT.

This study demonstrated a significant correlation between post-procedural ALFT and the severity of stroke. Similar to the findings of previous researches, subjects with ALFT were associated with higher NIHSS scores during hospitalization, indicating more severe stroke (15, 18). Analogously, Muscari et al. elucidated that impaired metabolic homeostasis in the liver was associated with cerebral infarct volume in experimental stroke models (27).

Inspiringly, this study revealed the reliable prognostic value of post-procedural ALFT on predicting functional outcomes of AIS patients treated with MT. This finding was in line with previous reports on the prognostic role of ALFT in AIS (15, 18). Substantial evidence expounded that hepatic dysfunction served as a predictor of clinical outcomes in critically ill patients (11–14). In addition, a population-based research indicated abnormal liver tests were associated with increased all-cause mortality in elderly people (28). However, in our study, data regarding liver function tests before the procedure were limited, and no significant association between preprocedural ALFT and the prognosis was observed. There might exist bias regarding preprocedural ALFT, as patients with severe ALFTs might not receive MT. This might partly explain why the prognostic value of post-procedural ALFT was important than the preprocedural ALFT. Furthermore, results of post-procedural liver function tests may be more representative of the physiological state and restoration of metabolic homeostasis.

Despite unclear specific mechanisms, it is conceivable that ALFT affects functional outcomes since the liver is an important organ for metabolism and immunity (29). It is known that AIS patients suffer from potential immunodepression due to the post-stroke autonomic system activation (30, 31). Subsequent infections and impaired metabolic homeostasis contribute to organ abnormalities, ultimately resulting in the poor prognosis of AIS (23).

Several strengths of our study were noteworthy. Relatively intact data from multiple centers revealed the contemporary status of MT for AIS-LVO in China. Moreover, comprehensive analyses were performed to explore the relationship between ALFT and functional outcomes after MT. Most importantly, liver function tests serve as routine examinations during hospitalization. The availability of test results makes its predictive value more significant for clinical application.

Despite the advantages mentioned previously, our study also had some inevitable limitations. First, this study is subject to the inherent limitations of a retrospective study design. Second, the liver function tests containing only four liver chemistries. Incomprehensive assessments made limited contributions to revealing the detailed mechanism. Third, missing records of the adjuvant medicine, especially for liver protection drugs, was another limitation. Finally, only the Chinese population was enrolled in this study, which may limit the generalizability of the conclusions. Further researches based on different ethnic populations and larger sample sizes are warranted.

## Conclusions

In this study, post-procedural ALFT occurred commonly in AIS patients treated with MT. It was associated with the severity of stroke and was an independent predictor for worse functional outcomes. More attention is needed for AIS patients who were diagnosed as ALFT after MT concerning the increased risk for poor prognosis.

## Data Availability Statement

The raw data supporting the conclusions of this article will be made available by the authors, without undue reservation.

## Ethics Statement

The studies involving human participants were reviewed and approved by The Ethics Committee of Jinling Hospital and each participating center. Written informed consent for participation was not required for this study in accordance with the national legislation and the institutional requirements.

## Author Contributions

RL, KH, and XL: concept and design. KH, MZ, MW, and QY: acquisition of data. KH, MZ, RL, and LX: analysis and interpretation of the data. KH, MZ, LX, JG, and JD: drafting of the article. RL, KH, MZ, and XL: critical revision of the article for important intellectual content. All authors contributed to the article and approved the submitted version.

## Conflict of Interest

XL served as the principal investigator for the Captor clinical trial sponsored by Shanghai strokecare medical Co. LTD. RL served as a researcher for the Captor clinical trial and provided consultation to Shanghai strokecare medical Co. LTD. The remaining authors declare that the research was conducted in the absence of any commercial or financial relationships that could be construed as a potential conflict of interest.

## Publisher's Note

All claims expressed in this article are solely those of the authors and do not necessarily represent those of their affiliated organizations, or those of the publisher, the editors and the reviewers. Any product that may be evaluated in this article, or claim that may be made by its manufacturer, is not guaranteed or endorsed by the publisher.
