# Supplementary figures and images for: Prognostic Value of Abnormal Liver Function Tests After Mechanical Thrombectomy for Acute Ischemic Stroke
Source: Front Neurol. 2021 Jul 28;12:670387. doi: 10.3389/fneur.2021.670387 (PMC8356900; doi:10.3389/fneur.2021.670387)

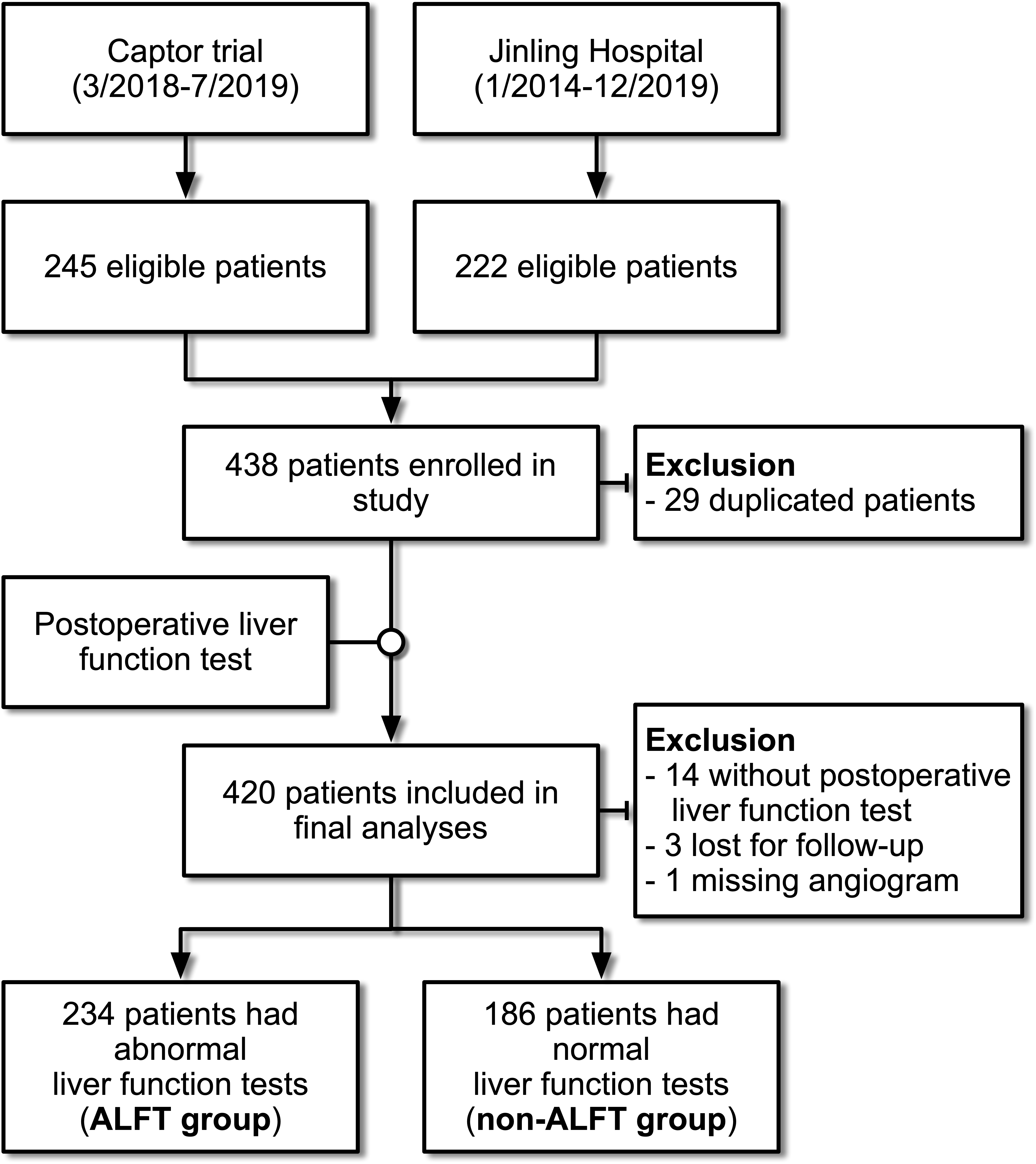

Supplement: Supplementary file 1 [file Image_1.TIFF]

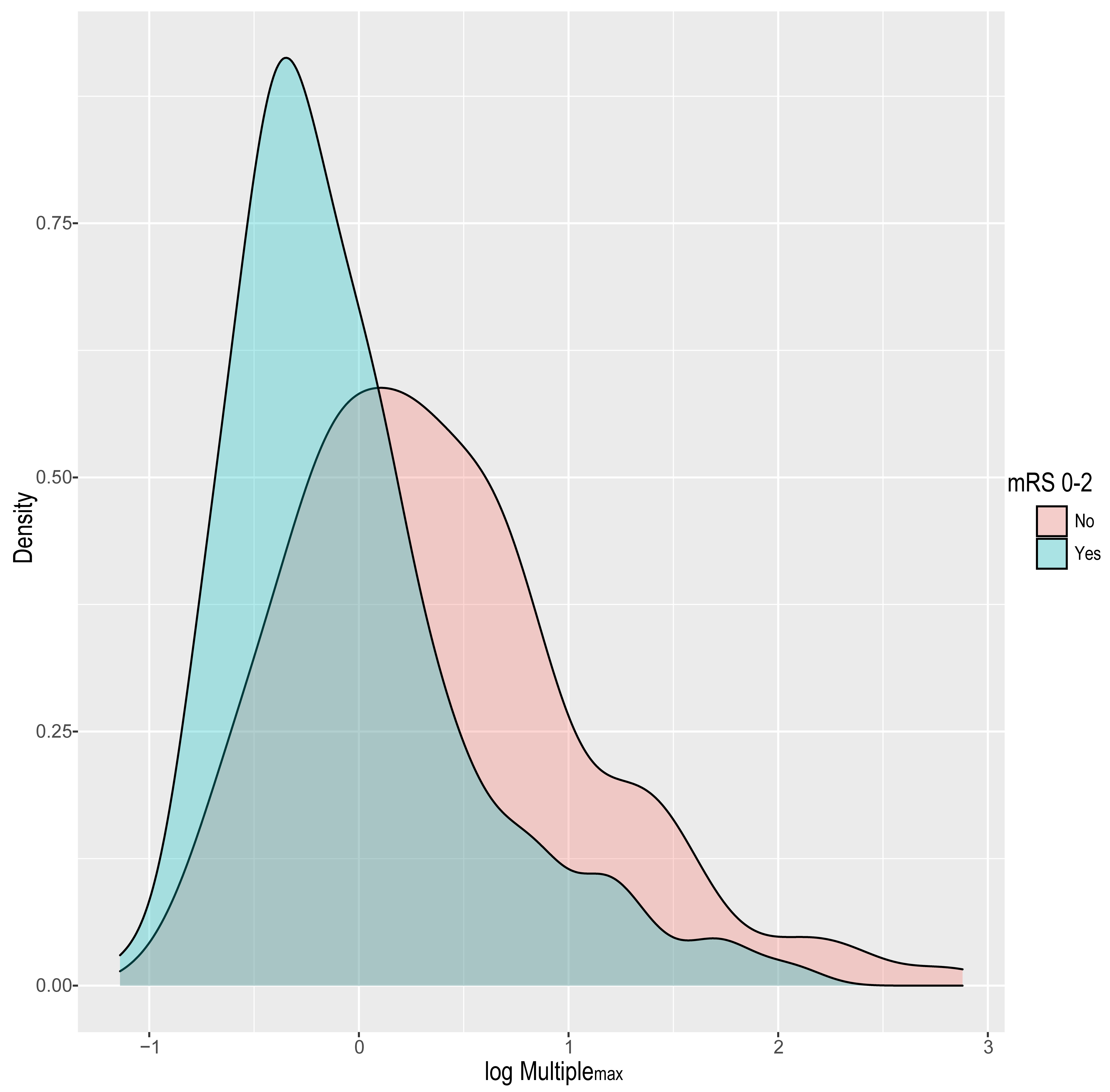

Supplement: Supplementary file 2 [file Image_2.TIFF]
