# Supplementary material for: Prognostic Value of Abnormal Liver Function Tests After Mechanical Thrombectomy for Acute Ischemic Stroke
Source: Front Neurol. 2021 Jul 28;12:670387. doi: 10.3389/fneur.2021.670387 (PMC8356900; doi:10.3389/fneur.2021.670387)
Supplement: Supplementary file 3 [file Data_Sheet_1.docx]

**Supplementary Figure 1**. Flowchart of this study.

Abbreviations: NSRP, the Nanjing Stroke Registry Program; ALFT, abnormal liver function test.

**Supplementary** **Figure 2**. Density distribution plots of *log*-transformed Mulitple_max_ in patients with different prognosis.

Density distribution plots of *log*-transformed Mulitple_max_ in patients with favorable prognosis (90-day modified Rankin Scale [mRS] score 0-2, in green color) and non-favorable prognosis (90-day mRS 3-6, in pink color).

Abbreviations: mRS, modified Rankin scale.
